# Mössbauer and X-ray Diffraction Spectroscopy of High-Iron Bauxites from Kazakhstan

**DOI:** 10.3390/ma16206706

**Published:** 2023-10-16

**Authors:** Adilkhan Shokanov, Mikhail Vereshchak, Irina Manakova, Anastassiya Migunova

**Affiliations:** 1 Abai Kazakh National Pedagogical University, Dostyk Av. 13, Almaty 050010, Kazakhstan; 2 Institute of Nuclear Physics, Ibragimov St. 1, Almaty 050032, Kazakhstan; mikhail.vereshchak@mail.ru (M.V.);; 3 Al-Farabi Kazakh National University, Al-Farabi Av. 71, Almaty 050040, Kazakhstan

**Keywords:** bauxite, Mössbauer spectroscopy, X-ray diffraction

## Abstract

The bauxite ores of Kazakhstan were analyzed using Mössbauer spectroscopy, X-ray diffraction and an X-ray fluorescence analysis. Experimental data on the structural–phase composition of bauxites were obtained, and the features of the iron-bearing minerals within them were revealed. The studied bauxites were high in iron. The magnetic part of bauxite was mainly represented by aluminohematite with a concentration of C_Al_ = 3.34–5.73 at.%, alongside goethite in small amounts. The predominant phase in the bauxite samples was the alumina-bearing mineral gibbsite with a well-crystallized monoclinic lattice. The main siliceous mineral of bauxite is kaolinite, which showed distorted octahedral positions in a number of samples. Siderite amounts were found to vary in the range of 0–15 at.% in the present iron-bearing minerals. Ilmenite was also present in the bauxite of some deposits; anatase was found in all bauxites and was the final product of ilmenite decomposition in the weathering crust.

## 1. Introduction

Aluminum is one of the most in-demand metals in the world. It is often used in aviation, shipbuilding, automotive, energy, architecture and other industries. The most important aluminum ore is bauxite in the production of alumina. The high rate of aluminum consumption requires a continuous increase in bauxite production, which currently equals 4–5% per year. This trend could lead to the depletion of high-grade bauxite reserves. In addition, high-quality bauxite reserves are limited in many countries. The growing need for aluminum in such conditions can be satisfied by involving low-grade, high-iron bauxite in processing. In this regard, studies on iron compounds in bauxite and their influence on the technological process of alumina production are relevant.

Bauxite contains up to 100 elements in the Periodic Table with various combinations. The number of minerals is also close to 100. From a technological point of view, all bauxite minerals can be divided into three groups. The first group includes aluminum-containing minerals: gibbsite, boehmite and diaspore. The second group includes minerals that impede or disrupt the technology of alumina production. These are silica-containing minerals, silicates and aluminosilicates, sulfides, carbonates and organic substances. The third group includes ballast compounds, which remain unchanged during technological processing and are removed from the technological cycle in the form of sludge. These are iron oxides and titanium-containing compounds. The content of silica (SiO_2_) in bauxite varies over a wide range (2–20%) and is characterized by a silicon modulus μ_Si_ = Al_2_O_3_(%)/SiO_2_ (%). A higher silicon module is considered an indicator of a better quality of bauxite. If μ_Si_ ≥ 6–8, such bauxites can be processed according to the Bayer method [1,2,3]. If μ_Si_ < 6–8, the sintering method is used [2]. The main silica-bearing mineral in bauxite is kaolinite. Quartz is usually included in bauxite in the form of an α-modification: α-SiO_2_. Its content in bauxite ranges from 3 to 11%. Iron compounds are present in bauxites, along with the main rock-forming minerals, oxides and hydroxides of aluminum. These include oxides, sulfates and sulfides, as well as silicates and carbonates. Hematite and hydrohematite, goethite and hydrogoethite, limonite and hematogel, as well as magnetite and maghemite, can be distinguished among these oxides. Sulfides are represented by pyrite, melnikovite-pyrite, chalcopyrite, marcasite and pyrrhotite. Gibbsite bauxites also contain carbonates, with the most common being FeCO_3_ siderite. This mineral is an extremely harmful impurity since it actively and irreversibly interacts with alkaline solutions, which leads to their decaustication. The main titanium-containing minerals are anatase, rutile, ilmenite and brookite; less often, sphene, titanomagnetite and perovskite are also found.

Kirwan et al. [4] characterized various Jamaican bauxite ores using X-ray powder diffraction (XRD) and Mössbauer spectroscopy (MS). The main iron minerals found were crystalline hematite and crystalline aluminogethite, together with small-sized crystallite. In [5], minerals in Jamaican bauxite were studied using powder X-ray diffraction and nuclear magnetic resonance. Gibbsite, boehmite, hematite, aluminous goethite and anatase were identified in both hematite-rich and goethite bauxites. The results of mineral–crystallochemical studies on Timan ferruginous bauxites were presented in [6,7,8]. The features of distributing Fe^2+^ and Fe^3+^ ions over structural positions in oxides and phyllosilicates were also discussed. In this study, the phase composition of ferruginous bauxites was represented by boehmite, hematite, ultrafine, low-ordered goethite and berthierine. The structural impurity of iron up to 10% and an admixture of aluminum in iron hydroxides were found in boehmite and kaolinite. MS and XRD studies on Salem bauxites, Tamil Nadu (India), were presented in [9]. This analysis showed the predominant presence of Al-substituted goethite and hematite. Considerably small amounts of magnetite and ilmenite were also found. Two batches of bauxite samples from the mines in northern Brazil, as well as from the Kibi deposit in Ghana, were analyzed in [10]. Attention was paid to the isomorphic substitution of aluminum for iron in hematite. The aluminum content in hematite ranged from 5.7 to 12.2 mol %.

A large number of bauxite deposits are concentrated in the territory of Kazakhstan. Kazakhstan ranks twelfth in the world from the perspective of bauxite reserves. Their deposits are grouped in several bauxite-bearing areas. The largest of them is West Torgaiskoye, which includes the Krasnooktyabrskoye bauxite-bearing zone, which holds 85.2% of Kazakhstan’s balance reserves. The largest deposits in this zone are Ayatskoye, Krasnooktyabrskoye, Belinskoye and East Ayatskoye, with significant resources predicted (50–100 million tons). However, the bauxite in the Krasnooktyabrskoye bauxite-bearing zone contains a high number of harmful impurities (Fe, Si, C, etc.). Constant research and development is required to improve the methods for extracting alumina from feedstock based on real circumstances. Despite the attempts of earlier studies on the phase composition of bauxite in Kazakhstan using mineralogical, X-ray phase and thermal analyses [11,12,13], no special attention has been paid to the study of iron-bearing minerals.

Currently, a number of methods are used to study bauxite. One of the most common is X-ray diffraction. This method identifies mineral phases and determines the atomic structure of minerals. The full profile Rietveld method seems to be a reliable method in the study of bauxite ores [4,14,15].

In nature, bauxite contains iron, which allows a very effective and convenient method of nuclear gamma-resonance spectroscopy on ^57^Fe nuclei to be used—also known as the Mössbauer effect [16,17,18]—to study the mineral composition of alumina feedstock. This is associated with iron’s unique ability to act as an isomorphic impurity in a wide range of minerals. In addition, iron is the basis of a number of natural resources (iron oxides and hydroxides, carbonates, sulfates, silicates, titanates and others). Mössbauer spectroscopy provides information about the crystal and chemical features of iron ions in the structure of minerals and distinguishes impure iron from oxide iron in magnetically ordered oxides [19,20,21,22,23,24]. Thus, Mössbauer spectroscopy makes evaluating the iron ore composition of bauxites possible at all stages of the industrial production of alumina. In particular, useful information can be obtained using MS and XRD in combination with an X-ray fluorescence analysis (XRF).

The purpose of this research is to obtain reliable experimental data on the structural–phase composition of bauxite ores in Kazakhstan and identify the features of iron-bearing minerals in bauxites of various deposits using the MS, XRD and XRF methods.

## 2. Materials and Methods

Samples of bauxite from the Ayatskoye, Krasnooktyabrskoye, Belinskoye and East Ayatskoye deposits (the Krasnooktyabrskoye bauxite-bearing zone of the West Torgaiskoye bauxite-bearing region) were studied in this paper. Bauxite from the Arkalykskoye deposit (the Arkalyk bauxite-bearing zone of the Amangeldy bauxite-bearing region) was analyzed for comparison. The processing of bauxite from the Arkalykskoye deposit began a long time ago and has now stopped. Bauxite samples from this deposit were collected from a depth of 25 m (A1) and from the surface (A2). The bauxite in these deposits is of the gibbsite type according to its mineralogical features.

The elemental composition of powder samples was determined on an X-ray fluorescence energy-dispersive spectrometer RLP-21 with a semiconductor detector. The RLP-21 spectrometer provides a quantitative analysis for a range of elements from Al to U at concentrations from 0.001 to 100% for the middle range of elements and from 0.1% for light elements. This work was performed according to the methodology “Determination of the elemental composition of powder samples” developed for these deposits.

XRD was used to identify the mineral phases of the studied bauxite. The measurements were performed on a D8 ADVANCE universal complex (Bruker, Germany) using a tube with a copper anode (wavelength is 1.5406 Å) alongside operating parameters of 40 kV, 40 mA and 2θ in the range of 10–70° on the tube. Qualitative phase analysis was performed using the EVA program. The ICDD (International Center of Diffraction Data Database (PDF2 powder database)) was used to determine the indices (hkl) of diffraction reflections from the crystallographic planes of the studied samples.

The chemical state of iron in the crystal and magnetic structure of iron-bearing bauxite-forming minerals was studied using Mössbauer spectroscopy.

Mössbauer measurements were performed on an MS-1104Em spectrometer at room temperature in transmission geometry with a moving absorber. Mössbauer spectra were recorded on 1024 channels in the constant acceleration mode with a triangular law of velocity variation in the range of ±10 mm/s. The counting rate was 50–60 thousand pulses/channel/hour. Subsequently, folding was used to obtain the 512-channel spectrum. ^57^Co in a chromium matrix with an activity of 25 mCi (Cyclotron Co. Ltd., Obninsk, Russia) was used as a source of γ-quanta; a resonant scintillation detector was used for the registration of Mössbauer spectra. The use of resonant detectors is one of the ways to increase the productivity of the Mössbauer measurements [25,26,27,28] and has a number of advantages, such as improved resolution and increased peak intensity. The resonant detectors reduce the width of the experimentally observed spectral line to 1.43–1.46 Γ [26,27,28], where Γ is the line width of the excited-state nuclear level. It should be noted that, in the case of resonant detector applications, the shape of the spectrum line differs from the Lorentzian one [26,28]. It is necessary to take into account the spectral selectivity of the detector for spectra fitting. The Mössbauer spectra were processed using the SpectrRelax program [29]. The model-dependent method was used for the paramagnetic components of the studied materials. This method is based on individual components that can be related to the different compounds or local microenvironments. The restoration of distributions of hyperfine magnetic fields was used for the description of magnetic components. The criteria of the fitting quality were the differential spectrum, χ^2^, and the physical meaning of the parameters. The pseudo-Voigt function, a superposition of the Lorentzian and Gaussian functions, was chosen to fit the line shape. The following parameters were determined for each spectrum: isomer shift relative to metallic iron, δ, quadrupole splitting, Δ, quadrupole shift for magnetically split spectra, ε, magnetic hyperfine field, H, line width, Γ, and relative spectral area, A. An α-Fe foil of 30 μm thickness was used as the reference absorber. The width of the third and fourth lines of the spectrum was 0.21 mm/s. The studied sample was in the form of a tablet 20 mm in diameter. For its preparation, ~300 mg of bauxite ore ground in an agate mortar was mixed with paraffin and pressed into a tablet. Thus, the thickness of the samples was 20 mg Fe/cm^2^. Under conditions where the absorber thickness exceeds the thinness limit, line broadening will naturally occur, but the advantage of relatively narrower lines, compared to a conventional set-up, will remain [27]. The spectrum’s measurement time varied from 20 to 80 h. The instrumental error when estimating the values of hyperfine structure parameters did not exceed ±0.04 mm/s. After comparing the calculated and instrumental errors, the largest error value was given for parameter estimates.

## 3. Results

### 3.1. X-ray Fluorescence Analysis

The results of analyzing the elemental composition of the studied bauxite samples are summarized in Table 1. The XRF data of the main components were recalculated for oxides and reduced to 100%. The main components of the bauxites were Al_2_O_3_ and SiO_2_. In addition, the content of Fe_2_O_3_ was quite high and amounted to 24–33 wt. % for the bauxites of the Ayatskoye, Krasnooktyabrskoye, Belinskoye and East Ayatskoye deposits. The silicon module, μ_Si_, was determined. The table does not indicate chemical elements that had a content of less than 1 mg/g.

### 3.2. Mössbauer Spectroscopy

**Figure 1:**
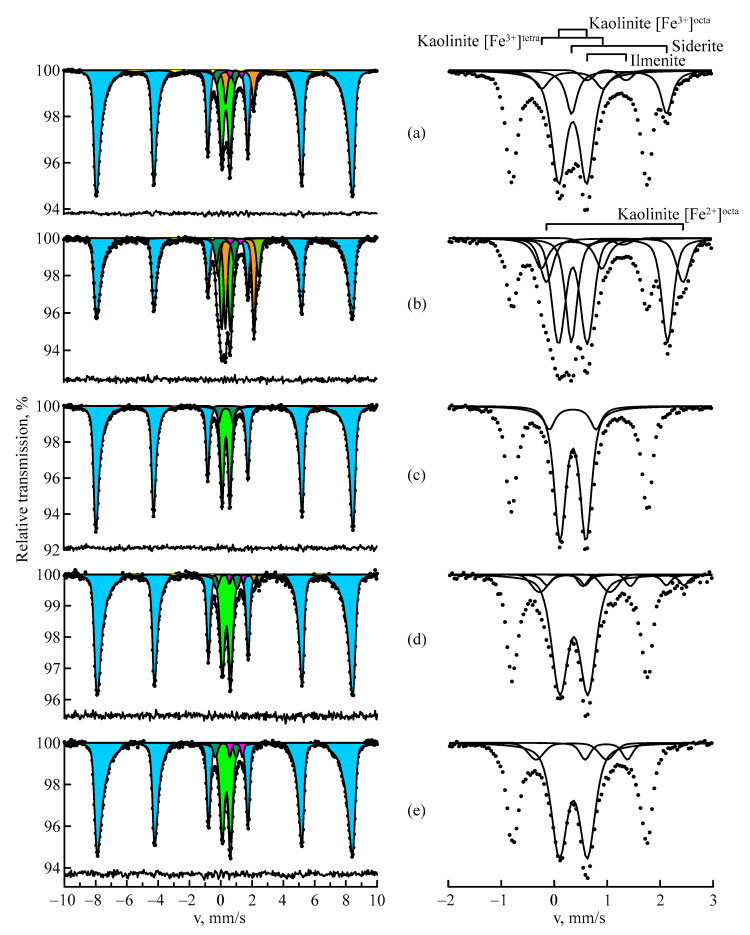
MS spectra on ^57^Fe nuclei of the bauxite samples from the Ayatskoye (**a**), Krasnooktyabrskoye (**b**), Belinskoye (**c**), East Ayatskoye (**d**) and Arkalykskoye A1 (**e**) deposits: hematite (blue), goethite (yellow), kaolinite (green, kentucky green and martian green), siderite (orange), ilmenite (magenta). Left panel with the Mössbauer spectra in velocity range ±10 mm/s and right panel with the enlarged central parts of the correspondent spectra in velocity range −2–+3 mm/s.

The Mössbauer spectra of the studied bauxite are shown in Figure 1. In Figure 1, there are two panels: the left panel with the Mössbauer spectra in the velocity range ±10 mm/s and the right panel with the enlarged central parts of the correspondent spectra in the velocity range −2–+3 mm/s. It can be seen that the iron in the studied bauxite was in magnetically ordered and paramagnetic states (Table 2). There are minor misfits at the residuals related to the resonant peaks of hematite. It can be assumed that this is associated with local heterogeneity in the nearest environment of iron atoms in Al-substituted hematite. The magnetic component of the MS spectra was predominantly represented by hematite, Fe_2_O_3_, and, in small amounts, by goethite, α-FeOOH. It can be seen that the hyperfine magnetic fields of Fe_2_O_3_ in the MS spectra for all studied bauxite turned out to be somewhat lower than the field of reference for Fe_2_O_3_ (515 kOe [16,30]). This could be explained by the presence of aluminum in the crystal lattice of hematite, which reduces the effective magnetic hyperfine fields on ^57^Fe nuclei [30,31]. As shown by Vandenberghe et al. [19], the dependence H_eff_ can be realized at room temperature in the concentration range of up to 16 at.% Al:

H_eff_ = 516.5 − 60.8x_Al_, (1)

where x_Al_ is the mole fraction of Al in Fe_2_O_3_.

Another Mössbauer parameter, the quadrupole shift, increased compared with the analogous parameter of the reference Fe_2_O_3_ (2ε = −0.17 mm/s) [16,30], which is associated with an increase in the distortion of the crystal lattice due to the incorporation of Al^3+^ into Fe^3+^ sites. 

It should be noted that iron oxides in the weathering crust are formed of fine particles. Thus, the predominant part of iron in the studied bauxite was represented by aluminum-bearing hematite. This negative effect of iron leads to irretrievable losses of alumina in the process of bauxite processing.

The paramagnetic part of the bauxite spectra has a rather complex structure and may include five doublets due to the presence of three minerals: kaolinite, siderite and ilmenite.

Five modifications of aluminum hydroxide (diaspore, boehmite, gibbsite, bayerite, nordstrandite) and one modification of aluminum oxide corundum have been found in nature. Natural aluminum hydroxides are formed in the following sequence: alumogel → boehmite → bayerite → gibbsite. The rate of this process depends on a number of factors (temperature, alkaline environment, etc.) and grows with their increase. One of the main minerals of bauxite is gibbsite, Al(OH)_3_. Up to a 2% concentration of iron can be present in gibbsite as an isomorphic impurity [2]. However, the parameters of MS spectra confirming the impurity of iron in gibbsite could not be found in the literature.

The main silicon-bearing mineral of bauxite is kaolinite, Al_2_Si_2_O_5_(OH)_4_. Quite often, kaolinite crystals are covered with a film of iron hydroxide. The MS spectrum of kaolinite is a superposition of three doublets, which characterizes three states of iron in the crystal lattice of the mineral: [Fe^3+^]^octa^-, [Fe^3+^]^tetra^- and [Fe^2+^]^octa^-positions [6,20,32]. The predominant part of iron in kaolinite is formed in the trivalent state, populating mainly octahedral sites. The Mössbauer parameters of [Fe^3+^]^octa^, as indicated in [20,33], are the most reliable: δ = 0.35 mm/s and Δ = 0.51 mm/s. As can be seen from Table 2, in the presence of Fe^2+^, the quadrupole splitting of Fe^3+^ was greater than the above value. This could be explained by the fact that the ionic radius of Fe^2+^ (0.78) is larger than the ionic radius of Fe^3+^ (0.55). This leads to a distortion in the octahedral positions. The state of Fe^2+^ in the composition of kaolinite is unstable, and it is not present in the bauxite from the Belinskoye and Arkalykskoye deposits. The isomer shift of [Fe^3+^]^tetra^ differs from that indicated in [32]. Most likely, this is due to the fact that kaolinite is present in the bauxite of Kazakhstan in an insufficiently crystallized state. In addition, the low iron content in kaolinite can lead to slow paramagnetic relaxation [20].

The fourth doublet of the MS spectrum was represented by the most abundant mineral in gibbsite bauxites—iron carbonate—siderite, FeCO_3_. Its content varied widely in these bauxites. Thus, its content was the highest (15.0 at. %) in the bauxite from the Krasnooktyabrskoye deposit, 1.2 at. % in the East Ayatskoye deposit, and FeCO_3_ was not present in deposits from Belinskoye and Arkalykskoye. Siderite is a very harmful impurity since it actively and irreversibly interacts with alkaline solutions, leading to their decaustication [2].

The fifth doublet corresponded to ilmenite, a titanium–iron mineral of FeTiO_3_. This mineral is relatively stable in the weathering crust. However, the content of ilmenite decreases more along the section of the weathering crust profile. This effect was convincingly demonstrated in bauxite from the Arkalykskoye deposit, collected from a depth of 25 m (A1) and the near-surface layer (A2). Fe^2+^ in the ilmenite lattice is oxidized to Fe^3+^, and the remaining titanium dioxide is concentrated in the form of leucosene—rutile—anatase. The decomposition of ilmenite occurs in the weathering crust in the direction of ilmenite—leucosenized ilmenite—leucosene—rutile—anatase. It should be noted that a typical phenomenon in bauxites is the process of component transfer from one phase to another or within one phase in the direction of the component’s decrease in concentration. A good example of this is the aforementioned decay of ilmenite.

Table 2 shows that the Fe_2_O_3_ content in bauxite from the Krasnooktyabrskoye deposit was 54.1 at. %. Annealing a sample of this bauxite at a temperature of 600 °C leads to the decomposition of goethite, siderite and ilmenite and the formation of hematite; Fe^2+^ in kaolinite is oxidized to Fe^3+^ with the formation of this oxide. Thus, the content of Fe_2_O_3_ increases to 84.2 at. % as a result of thermal treatment.

### 3.3. X-ray Diffraction

**Figure 2:**
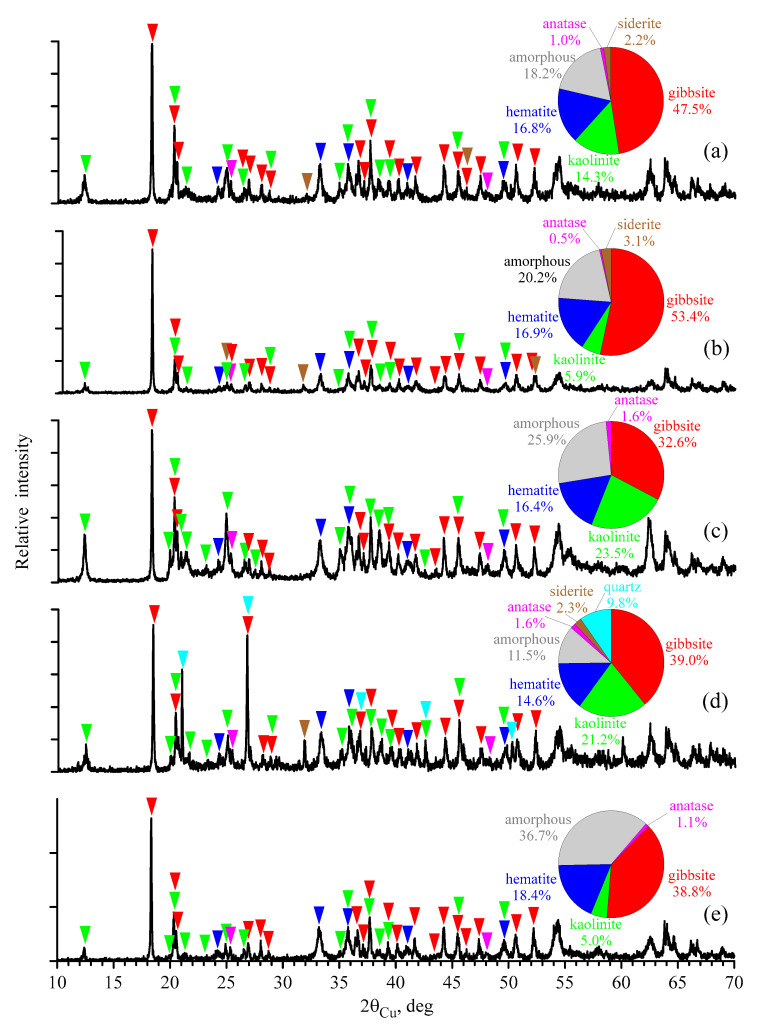
X-ray diffraction patterns of the bauxite samples from the Ayatskoye (**a**), Krasnooktyabrskoye (**b**), Belinskoye (**c**), East Ayatskoye (**d**) and Arkalykskoye A1 (**e**) deposits.

Figure 2 shows the XRD patterns of the studied bauxite. The dominant phase is gibbsite Al(OH)_3_ with a well-crystallized monoclinic lattice in the bauxite samples from all deposits.

The content of hematite, Fe_2_O_3_, with trigonal syngony is significant. In addition, the Fe_2_O_3_ reflections in all samples shift toward larger angles, i.e., a decrease in the interplanar distances is observed. Table 3 shows the parameters for the crystal lattice of hematite. As can be seen, parameter *a* is reduced compared to the reference (5.034); parameter *c* is also less than the reference (13.746) in most samples. Like the decrease in the hyperfine magnetic field in MS spectra, the decrease in the crystal lattice parameters of hematite is associated with the presence of aluminum in the lattice of this mineral. Since the Al^3+^ ion is smaller than the Fe^3+^ ion, when iron is replaced by aluminum in the Fe_2_O_3_ structure, the average unit cell size decreases. To estimate the degree of substitution, it is convenient to use reflection (104)—2.698 Å. The width of this line is the maximum on the X-ray diffraction patterns of bauxite from the Arkalykskoye deposit A1, which indicates a high degree of crystal lattice distortion and correlates with the Al concentration in Fe_2_O_3_ determined by the Mössbauer studies (see Table 2). The higher the degree of Al^3+^ substitution in hematite, the smaller the size of its crystallites and the lower the degree of their crystallization and order [4].

The hyperfine field of hematite at room temperature is a function of at least two parameters: the substitution of aluminum for iron and particle size [31]. Kirwan et al. [4] believe that the spectrum of goethite shows a doublet at a particle size less than 10 nm, while hematite exhibits paramagnetism at a particle size of 7 nm. With an average particle size of ~60 nm, hematite shows a sextet with a magnetic field that experiences only a slight deviation from the reference. Table 4 shows the estimated average particle sizes of the hematite contained in the bauxite samples, determined via MS and XRD methods. The size of the crystallites based on the XRD results was determined using the Scherrer formula [34], which takes into account the broadening of diffraction reflections associated only with size effects. The size of the hematite particles based on the MS results was determined using the distribution of magnetic fields.

The clay mineral kaolinite with the triclinic system Al_2_Si_2_O_5_(OH)_4_ can also be referred to as the main minerals of bauxite. Its amount was relatively large in the bauxite from the Belinskoye deposit. The X-ray diffraction patterns of the studied samples show traces of anatase, TiO_2_, with a tetragonal crystal lattice. Anatase is the final decomposition product of ilmenite, FeTiO_3_, in the weathering crust. Ilmenite is a finely dispersed material and, therefore, is not available for XRD. Siderite, FeCO_3_, with trigonal syngony, is also present in the bauxites of the Ayatskoye, Krasnooktyabrskoye and East Ayatskoye deposits, which is consistent with the MS data.

All the bauxites also contained some amount of the amorphous phase. The blurring of the reflections and an increase in the background of some areas of diffraction patterns at large angles could be explained by a set of the same phases, though of a nonstoichiometric composition, with a large set of inclusions.

## 4. Conclusions

An analysis of bauxite ores in Kazakhstan was performed to identify the features of bauxite from various deposits in order to propose adjustments to the technological process of alumina production. The MS, XRD and XRF methods were used to characterize the studied bauxites. Experimental data on the structural–phase composition of bauxite ores were obtained, and the features of iron-bearing minerals in them were revealed. It has been shown that the studied bauxite samples are high in iron. The magnetic part of bauxite is mainly represented by aluminohematite with a concentration of C_Al_ = 3.34–5.73 at.% and goethite in small amounts. The predominant phase in the bauxite samples was the alumina-bearing mineral gibbsite with a well-crystallized monoclinic lattice. The main siliceous mineral in bauxite was kaolinite, which showed distorted octahedral positions in a number of samples. Siderite amounts were found to vary in the range of 0–15 at.% for the iron-bearing minerals present. Ilmenite was also present in the bauxite of some of the deposits; anatase was found in all the bauxite samples, which was the final product of ilmenite decomposition in the weathering crust. The MS data correlated well with the XRD results and were consistent with the XRF.

The experimental data obtained could be useful for improving the technology used to process bauxite from specific deposits, taking into account their general physical–chemical properties and the individual differences in their mineral composition.

## Figures and Tables

**Table 1:** Chemical composition of bauxite.

Deposit	Main Components, wt. %						μ_Si_
	CaO	TiO_2_	Fe_2_O_3_	Al_2_O_3_	SiO_2_	K_2_O	
Ayatskoye	0.34	3.20	26.21	53.85	16.35	0.05	3.29
Krasnooktyabrskoye	0.86	3.45	30.15	50.81	14.70	0.03	3.46
Belinskoye	1.12	2.62	33.04	48.04	15.14	0.04	3.17
East Ayatskoye	2.18	3.20	24.14	53.48	16.68	0.32	3.21
Arkalykskoye (A1)	1.57	4.91	22.00	57.69	13.72	0.11	4.20
Arkalykskoye (A2)	0.11	3.25	18.91	56.43	14.13	0.17	3.99

**Table 2:** Mössbauer parameters of bauxite.

Deposit	State	A, %	δ, mm/s	Δ/2ε, mm/s	H, kOe	C_al_, at. %
Ayatskoye	Hematite	72.2 ± 0.1	0.37 ± 0.04	−0.22 ± 0.04	511 ± 1	3.34
	Goethite	2.3 ± 0.1	0.42 ± 0.04	−0.09 ± 0.04	364 ± 3	
	Kaolinite [Fe^3+^]^octa^	15.9 ± 0.2	0.36 ± 0.04	0.50 ± 0.04		
	Kaolinite [Fe^3+^]^tetra^	3.0 ± 0.2	0.35 ± 0.04	1.15 ± 0.04		
	Siderite	5.6 ± 0.1	1.23 ± 0.04	1.79 ± 0.04		
	Ilmenite	1.0 ± 0.1	1.00 ± 0.04	0.73 ± 0.04		
Krasnooktyabrskoye	Hematite	54.1 ± 0.3	0.37 ± 0.04	−0.21 ± 0.04	508 ± 1	5.12
	Goethite	1.4 ± 0.3	0.42 ± 0.04	−0.02 ± 0.04	370 ± 9	
	Kaolinite [Fe^3+^]^octa^	15.5 ± 0.3	0.36 ± 0.04	0.54 ± 0.04		
	Kaolinite [Fe^3+^]^tetra^	5.9 ± 0.3	0.34 ± 0.04	1.15 ± 0.04		
	Kaolinite [Fe^2+^]^octa^	7.2 ± 0.3	1.15 ± 0.04	2.58 ± 0.04		
	Siderite	15.0 ± 0.4	1.24 ± 0.04	1.82 ± 0.04		
	Ilmenite	1.1 ± 0.1	0.97 ± 0.04	0.67 ± 0.04		
Belinskoye	Hematite	78.3 ± 0.3	0.37 ± 0.04	−0.22 ± 0.04	510 ± 1	3.93
	Goethite	1.1 ± 0.3	0.42 ± 0.04	−0.08 ± 0.05	364 ± 8	
	Kaolinite [Fe^3+^]^octa^	17.2 ± 0.3	0.36 ± 0.04	0.49 ± 0.04		
	Kaolinite [Fe^3+^]^tetra^	3.5 ± 0.4	0.36 ± 0.04	0.88 ± 0.04		
East Ayatskoye	Hematite	74.2 ± 0.4	0.37 ± 0.04	−0.22 ± 0.04	508 ± 1	5.12
	Goethite	1.0 ± 0.4	0.42 ± 0.04	0.06 ± 0.06	360 ± 9	
	Kaolinite [Fe^3+^]^octa^	19.0 ± 0.6	0.37 ± 0.04	0.53 ± 0.04		
	Kaolinite [Fe^3+^]^tetra^	2.4 ± 0.3	0.39 ± 0.04	1.33 ± 0.04		
	Kaolinite [Fe^2+^]^octa^	1.3 ± 0.5	1.16 ± 0.04	2.59 ± 0.04		
	Siderite	1.2 ± 0.2	1.35 ± 0.04	1.53 ± 0.04		
	Ilmenite	0.9 ± 0.2	0.99 ± 0.04	0.90 ± 0.04		
Arkalykskoye (A1)	Hematite	75.8 ± 0.2	0.37 ± 0.04	−0.22 ± 0.04	507 ± 1	5.73
	Kaolinite [Fe^3+^]^octa^	19.5 ± 0.9	0.36 ± 0.04	0.51 ± 0.04		
	Kaolinite [Fe^3+^]^tetra^	2.7 ± 0.8	0.33 ± 0.04	1.34 ± 0.04		
	Ilmenite	2.0 ± 0.3	0.98 ± 0.04	0.80 ± 0.04		
Arkalykskoye (A2)	Hematite	73.7 ± 0.2	0.37 ± 0.04	−0.21 ± 0.04	508 ± 1	5.12
	Goethite	2.2 ± 0.2	0.42 ± 0.04	−0.12 ± 0.04	374 ± 2	
	Kaolinite [Fe^3+^]^octa^	18.7 ± 0.3	0.36 ± 0.04	0.51 ± 0.04		
	Kaolinite [Fe^3+^]^tetra^	3.8 ± 0.3	0.35 ± 0.04	1.05 ± 0.04		
	Ilmenite	1.5 ± 0.1	0.97 ± 0.04	0.69 ± 0.04		

C_al_: concentration of Al in Fe_2_O_3_.

**Table 3:** Parameters of the Fe_2_O_3_ lattice.

Deposit	*d*_(104)_	*d*_(110)_	*d*_(300)_	*a*, Å	*c*, Å
Ayatskoye	2.698	2.513	1.453	5.033	13.736
Krasnooktyabrskoye	2.695	2.510	1.453	5.028	13.677
Belinskoye	2.696	2.511	1.453	5.027	13.746
East Ayatskoye	2.690	2.505	1.451	5.023	13.727
Arkalykskoye (A1)	2.693	2.510	1.452	5.020	13.719
Arkalykskoye (A2)	2.689	2.505	1.450	5.022	13.726

*d*: interplanar spacing; *a*, *c*: lattice parameters.

**Table 4:** Average particle sizes of Fe_2_O_3_ determined by MS and XRD methods.

Deposit	Average Particle Sizes, nm	
	XRD	MS
Ayatskoye	29	32
Krasnooktyabrskoye	33	29
Belinskoye	31	36
East Ayatskoye	26	28
Arkalykskoye (A1)	24	24
Arkalykskoye (A2)	32	26

## Data Availability

The data presented in this study are available on request from the corresponding author.

## References

[1] Habashi F., Donaldson D., Raahauge B. (2017). A hundred years of the Bayer process for alumina production. Essential Readings in Light Metals.

[2] Loginova I.V., Kyrchikov A.V., Penyugalova N.P. (2015). Alumina Production Technology.

[3] Shoppert A., Valeev D., Loginova I., Pankratov D. (2023). Low-temperature treatment of boehmitic bauxite using the Bayer reductive method with the formation of high-iron magnetite concentrate. Materials.

[4] Kirwan L.J., Deeney F.A., Croke G.M., Hodnett K. (2009). Characterisation of various Jamaican bauxite ores by quantitative Rietveld X-ray powder diffraction and ^57^Fe Mössbauer spectroscopy. Int. J. Miner. Process..

[5] Young N.J., Coley M.D., Greenaway A.M. (2019). Mineralogical investigations of Jamaican hematite-rich and goethite-rich bauxites using XRD and solid state ^27^Al and ^31^P MAS NMR spectroscopy. J. Geochem. Explor..

[6] Kotova O., Silaev V., Lutoev V., Vakhrushev A. (2016). Mineralogy and crystal chemistry of iron in the Timan bauxite and products of their technological processing. IOP Conf. Ser. Mater. Sci. Eng..

[7] Shmakova A., Kanev B., Gömze A.L., Kotova O. (2017). Crystal chemical characteristics and physical properties of ferrous minerals as the basis for the formation of functional materials. IOP Conf. Ser. Mater. Sci. Eng..

[8] Kotova O.B., Sun S., Razmyslov I.N., Simakova Y.S., Bezaeva N.S., Gomes Coe H.H., Nawaz M.F. (2023). High-Iron Bauxites: Composition Features and Processing Technology (The Middle Timan). Springer Proceedings in Earth and Environmental Sciences.

[9] Raj D., Harchand K.S., Maini V. (1993). Characterization of iron minerals in bauxite. Nucl. Instrum. Methods Phys. Res. Sect. B.

[10] Neumann R., Avelar A.N., Da Costa G.M. (2014). Refinement of the isomorphic substitutions in goethite and hematite by the Rietveld method, and relevance to bauxite characterisation and processing. Miner. Eng..

[11] Biryukova A.A., Dzhienalyev T.D., Tikhonova T.A. (2017). Ceramic Proppants Based on Kazakhstan Natural Alumosilicate Resources. Refract. Ind. Ceram..

[12] Abdulvaliyev R.A., Gladyshev S.V., Pozmogov V.A., Kasymzhanova A.K. (2019). Hydrochemical technology for processing the ferrous fraction of bauxites. Obogashchenie Rud.

[13] Biryukova A.A., Dzhienalyev T.D. (2019). Ceramic proppants based on high-ferrous bauxite. Int. J. Adv. Sci. Eng. Inf. Technol..

[14] Yuan S., Xiao H., Yu T., Li Y., Gao P. (2020). Enhanced removal of iron minerals from high-iron bauxite with advanced roasting technology for enrichment of aluminum. Powder Technol..

[15] Gasparini A.S., Fontes M.P.F., Pacheco A.A., Ker J.C. (2022). Gibbsite Crystallinity and Morphology in Ferralsols and Bauxites. Minerals.

[16] Goldanskii V., Herber R. (1968). Chemical Applications of Mössbauer Spectroscopy.

[17] Gütlich P., Bill E., Trautwein A.X. (2011). Mössbauer Spectroscopy and Transition Metal Chemistry: Fundamentals and Applications.

[18] Gonser U. (2014). Mössbauer Spectroscopy II.

[19] Vandenberghe R.E., Barrero C.A., Da Costa G.M., Van San E., De Grave E. (2000). Mössbauer characterization of iron oxides and (oxy)hydroxides: The present state of the art. Hyperfine Interact..

[20] Murad E., Fabris J.D. (2010). Kaolin mining and beneficiation: The role of iron. J. Phys. Conf. Ser..

[21] Radoń A., Łoński S., Kądziołka-Gaweł M., Gębara P., Lis M., Łukowiec D., Babilas R. (2020). Influence of magnetite nanoparticles surface dissolution, stabilization and functionalization by malonic acid on the catalytic activity, magnetic and electrical properties. Colloids Surf. A Physicochem. Eng. Asp..

[22] Shoppert A., Valeev D., Diallo M.M., Loginova I., Beavogui M.C., Rakhmonov A., Ovchenkov Y., Pankratov D. (2022). High-Iron Bauxite Residue (Red Mud) Valorization Using Hydrochemical Conversion of Goethite to Magnetite. Materials.

[23] Shokanov A., Vereshchak M., Manakova I. (2020). Mössbauer and X-ray Studies of Phase Composition of Fly Ashes Formed after Combustion of Ekibastuz Coal (Kazakhstan). Metals.

[24] Vereshchak M., Shokanov A., Manakova I. (2021). Mössbauer studies of narrow fractions of fly ash formed after combustion of Ekibastuz coal. Materials.

[25] Mashlan M., Kholmetskii A., Yevdokimov V., Pechousek J., Verich O., Zboril R., Tsonchev R. (2006). Mössbauer spectrometer with resonant detector. Nucl. Instrum. Methods Phys. Res. Sect. B.

[26] Mitrofanov K.P., Gor’kov V.P., Plotnikova M.V., Reiman S.I. (1978). Determination of the Mössbauer effect probability using resonance detectors. Nucl. Instrum. Methods.

[27] Odeurs J., Hoy G.R., L’abbé C. (2000). Enhanced resolution in Mössbauer spectroscopy. J. Phys. Condens. Matter.

[28] Belyaev A.A., Volodin V.S., Irkaev S.M., Panchuk V.V., Semenov V.G. (2010). Application of resonant detectors in Mössbauer spectroscopy. Bull. Russ. Acad. Sci. Phys..

[29] Matsnev M.E., Rusakov V.S. (2012). SpectrRelax: An application for Mössbauer spectra modeling and fitting. AIP Conf. Proc..

[30] Kuzmann E., Nagy S., Vértes A. (2003). Critical review of analytical applications of Mössbauer spectroscopy illustrated by mineralogical and geological examples (IUPAC Technical Report). Pure Appl. Chem..

[31] Murad E., Cashion J. (2004). Mössbauer Spectroscopy of Environmental Materials and their Industrial Utilization.

[32] Casteleina O., Aldonb L., Olivier-Fourcadeb J., Jumasb J.C., Bonneta J.P., Blancharta P. (2002). ^57^Fe Mössbauer study of iron distribution in a kaolin raw material: Influence of the temperature and the heating rate. J. Eur. Ceram. Soc..

[33] St. Pierre T.G., Singh B., Webb J., Gilkes B. (1992). Mössbauer Spectra of Soil Kaolins from South-Western Australia. Clays Clay Miner..

[34] Singh A.K. (2005). Advanced X-ray Techniques in Research and Industries.

